# Differential manifestation of RONS and antioxidant enzymes in response to singular versus combinatorial stress in *Chironomus ramosus*

**DOI:** 10.1007/s44154-022-00077-8

**Published:** 2022-12-29

**Authors:** Pratibha Bomble, Bimalendu B. Nath

**Affiliations:** 1 Stress Biology Research Laboratory, Department of Zoology, Savitribai Phule Pune University, Pune, 411007 India; 2 MIE-SPPU Institute of Higher Education, Doha, Qatar

**Keywords:** Abiotic stressors, Aconitase, Multiple stress, Oxidative stress

## Abstract

In nature, organisms face multiple abiotic stress concurrently. Our previous study has indicated how threshold level of lethality depends on the type and combination of stressors. Many mechanisms exist by which organisms respond to stressors and maintain homeostasis. We examined the homeostatic pliability in an extremophilic oriental midge *Chironomus ramosus* larvae under various combinatorial stress conditions of desiccation (DS), heat (HS) and starvation (SS). Exposure to these stressors led to activation of a common response pathway of oxidative stress. Abundance of antioxidant enzymes like superoxide dismutase, catalase, glutathione reductase and glutathione peroxidase along with selective as well as stressor specific increase in total antioxidant capacity were reflected from the corresponding level of reactive oxygen and nitrogen species (RONS) in larvae exposed to various combinatorial stress. Additionally, we found stressor specific increment in lipid peroxidation level, protein carbonyl content and advanced oxidative protein products during the stress regime. Further investigation revealed a sharp decline in the activity of mitochondrial aconitase enzyme activity in response to abiotic stress induced oxidative stress. The combinatorial stressor specific comparative study based on biochemical and fluorescence based redox-endpoint assays confirmed that the generation of oxidative stress is the consequential convergent pathway of DS, HS and SS, but the quantum of RONS decides the redox potential of homeostatic response and survival rate.

## Introduction

Under natural conditions, environmental stress portrays a complex variable of multiple stressors, abiotic as well as biotic and quite often, organisms face several stressors at the same time. Manifestation of various stressors can be additive or combinatorial (Liess et al. 2016; Nõges et al. 2016; Gotcha et al. 2017; Karla et al. 2017; Galvez 2018). The concept of stress combination has been first floated amongst the plant stress biologists (Mooney et al. 1991; Mittler 2006). Although plethora of information is available for plants, attention has also been focused on combinatorial responses to abiotic stressors in animal taxa thriving in diverse environmental conditions (Kaunisto et al. 2016; Gotcha et al. 2017; Karla et al. 2017).

Multifarious nature of current environmental deterioration demands a shift of conventional stress-response research from single-stressor model to multi-stressor paradigm (Todgham and Stillman 2013; Gunderson et al. 2016). It is important to examine the co-variates which can influence how an organism responds either to multiple stressors acting concurrently or to combination of stressors which determines the organism’s threshold limit and homeostatic plasticity. Such studies are plentiful on plants and there exists knowledge gaps in animal groups when we explore the literature barring a few noteworthy reports (Kaunisto et al. 2016; Huang et al. 2019 and references therein).

Non-biting Chironomid midges are one of the most common and abundant group of aquatic insects (Ferrington 2008). Sensitivity of Chironomid midges to several ecological parameters makes them an important bioindicator of aquatic ecosystem (Nicacio and Juen 2015). Previously published work from our laboratory revealed that insects like Chironomid midges and Drosophilid flies respond to multiple stressors differently for their responses to individual stressor with reference to threshold levels of tolerance (Bomble and Nath 2019). In the light of this, we have chosen one of the well characterized physiologically amenable insects *Chironomus ramosus*, an Oriental non-biting midge (Order: Diptera; Family: Chironomidae), for the present study which has been reported as tolerant to many biotic and abiotic stressors (Hardikar and Nath 2001; Datkhile et al. 2009; Thorat and Nath 2010; Thorat et al. 2017; Bomble and Nath 2019).

Oxidative stress is a common consequence of many abiotic stress conditions, which triggers organism’s antioxidant defense mechanisms. All aerobic organisms possess several enzyme and non-enzyme antioxidants. Antioxidant enzymes, such as superoxide dismutase (SOD), catalase (CAT), glutathione peroxidase (GPx) and glutathione reductase (GR), play important role as scavengers of reactive oxygen species (ROS) (Castro et al. 2019). Generation of ROS is the primary biochemical signature of oxidative stress-response. Growing evidences indicate that ROS generation leads to oxidative stress and during the course of evolution, all organisms developed homeostatic mechanisms against individual as well as multiple stressors which are driven through oxidative stress (Xie et al. 2019; Chaitanya et al. 2016). Similar to ROS, reactive nitrogen species (RNS) are additional set of free radical as well as non-radical molecules, generated under stress conditions leading to nitrosative stress. These redox-sensitive molecules have gained importance in the contemporary stress-biology research (Saddhe et al. 2019; Ozcan and Ogun 2015). Unlike several literatures available in plants, redox regulation of reactive oxygen and nitrogen species (RONS) has remained largely unexplored in animals during multi-stress conditions (Turkan 2017). However, in recent times, RONS have been highlighted in biomedical research and implicated in pathophysiological conditions (Sardella et al. 2020, von Woedtke et al. 2019, Chaitanya et al 2016, Alhasawi et al 2019). RONS generation has also been shown as the most common factor irrespective of the type of stress. Production of ROS and RNS leads to successive downstream responses and their levels are regulated by various oxidants and antioxidants, often referred to as ‘redox homeostasis’ (Raja et al. 2017; Nadarajah 2020).

One of the metabolic indicators of oxidative stress is the loss of aconitase activity (Castro et al. 2019). The mitochondrial enzyme, aconitase is known to provide reliable estimate of steady state concentration of superoxide in the mitochondrial matrix (Gardner 2002). Aconitase plays an important role in the Krebs cycle in isomerization of citrate to isocitrate. Aconitase belongs to a family of iron-sulfur containing dehydratases, having cubane [4Fe–4S] centers. Among these, three iron atoms interact with cysteine residues, while the fourth iron, Fe_α_, catalyzes dehydration of citrate to form the intermediate cis-aconitate, and subsequently hydration of cis-aconitate occurs to form isocitrate. The fourth iron Feα exposed to solvent for catalytic activity are also susceptible to attack by superoxide which leading to oxidative inactivation of aconitase (Gardner and Fridovich 1991, Gardner et.al. 1995). Oxidative inactivation of aconitase has been reported as an index of increased ROS levels (Cantu et.al. 2009). This is the rationale of investigating catalytic status of aconitase in the present study in the backdrop of abiotic stressor induced oxidative stress.

Under prevailing scenario of global warming and climate change, extreme desiccation can lead to collapse of food webs of aquatic ecosystem (Ledger et al. 2012). As a result, aquatic fauna may experience nutrient limitation at the time of drought coupled with fluctuation of thermal regime. Interestingly, heat stress is usually accompanied by additional abiotic stressors, such as desiccation and nutritional limitations. Thus, we found a rationale to select three representative abiotic stressors, namely starvation, desiccation and heat for the current study and for investigating the effect exerted by individual stressor or by combined stress with an aim to investigate redox homeostasis of multi-stress generated RONS. In this present study, we have aimed to see how production of RONS during combinatorial stress response is countered by the Chironomid midge larvae which are otherwise known to be tolerant to many adverse environmental conditions and thereby maintain the homeostatic redox pool. We have also investigated other facets of oxidative stress *viz*., oxidative protein damage in larvae following stress treatment. Accordingly, the extent of lipid peroxidation, levels of advanced oxidative protein products (AOPP) and resultant protein carbonyl contents were also determined. Therefore, the present study is an integrated approach to understand whether the magnitude of oxidative stress depends on type and combination of environmental stressors or not.

## Results

### Impact of oxidative stress

**Fig. 1:**
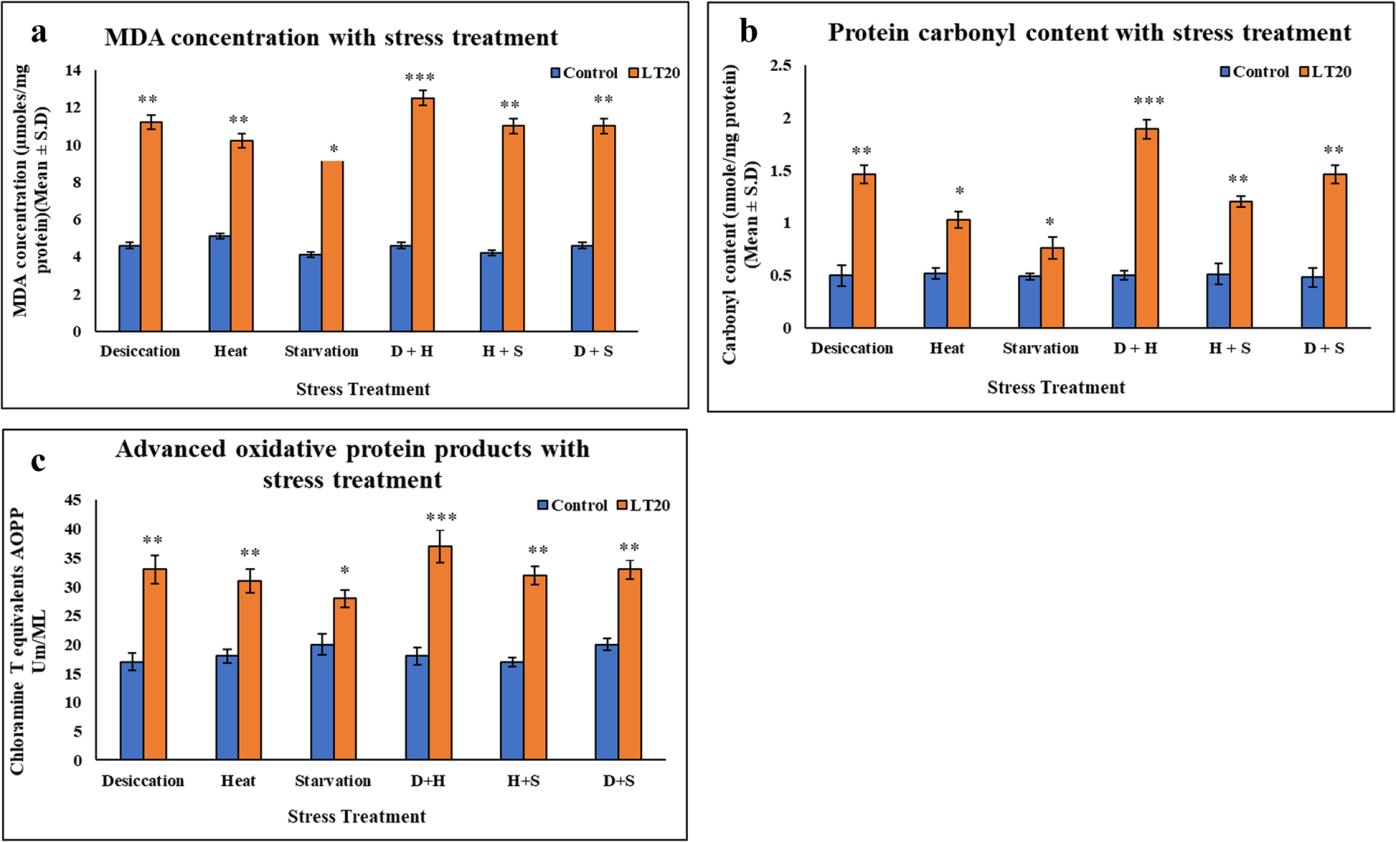
Impact of oxidative stress: Spectrophotometric measurement of **a** Lipid peroxidation (mean ± SD U/mg protein), MDA (malionaldehyde) concentration with stress treatment. **b** Protein carbonyl content (mean ± SD U/mg protein) **c** Advanced oxidative protein products (AOPP) (mean ± SD U/mL) in fourth instar larvae of *C. ramosus*. Values represent mean and the vertical bars represent SD. Data shown are representative of three independent experiments. ***P < 0.001; **P < 0.01; *P < 0.05 indicates level of significance

An increase in the concentration of malionaldehyde (MDA) was evident in treated larvae as compared to untreated larvae. The increase was more significant in the case of H+D than other combinatorial form of stressors. When present in singular form, it was more prominent in case of DS than the other two stressors (Fig.1a). There was increase in the level of protein carbonyl content with stress treatment but this increase was more prominent in case of DS than that of singular stressors like HS and SS. DS in combination with HS was found more vulnerable than the DS alone and also in other combinatorial forms (Fig.1b). Advanced oxidation protein products (AOPP) are toxins created during oxidative stress through the reaction of chlorinated oxidants with proteins. Here, there was significant increase in the concentration of AOPP in treated larvae compared to control larvae. This increase was more evident in combinatorial form (H+D) than in the form of singular stress exposure. Amongst the singular stressors, DS showed increased level of AOPP than the other two singular stressors (Fig. 1c).

### ROS generation

#### Measurement of superoxide radical and hydrogen peroxide

**Fig. 2:**
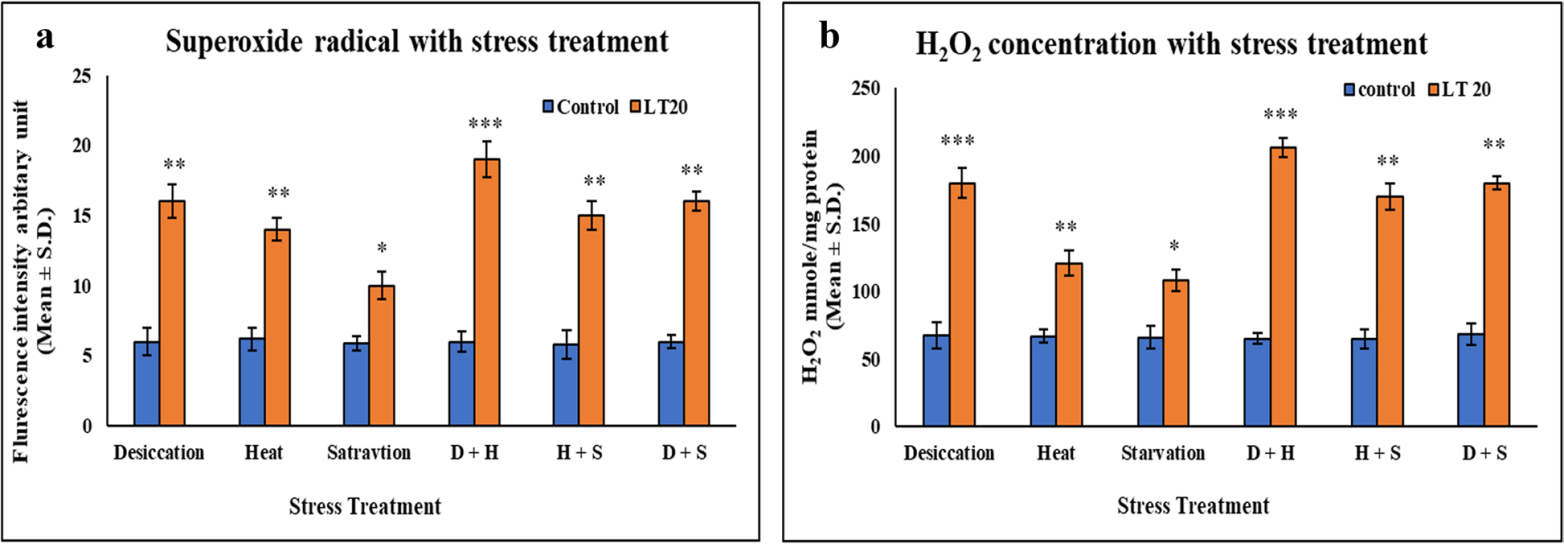
Spectrofluorimetric measurement of ROS **a** superoxide radical expressed as level of arbitrary unit (mean ± SD) **b** Quantification of Hydrogen peroxide (H_2_O_2_) nmole/mg of protein (mean ± SD) in fourth instar larvae of *C. ramosus*. Values represent mean and the vertical bars represent SD. Data shown are representative of three independent experiments. ***P < 0.001; **P < 0.01; *P < 0.05 indicates level of significance

Increment in ROS production, resulting from stress induced imbalance between oxidants and antioxidants was evident from the elevated level of superoxide radical and hydrogen peroxide subsequent to stress treatment as compared to control ones in larvae of *C. ramosus*. This increase was more visible during desiccation along with heat stress (D+H) than desiccation combined with starvation stress (D+S) and similar results were obtained in the case of heat combined with starvation stress (H+S). Also, in singular form, the increase was more prominent in the case of DS compared to HS and DS (Fig 2 a-b).

#### Imaging studies

**Fig. 3:**
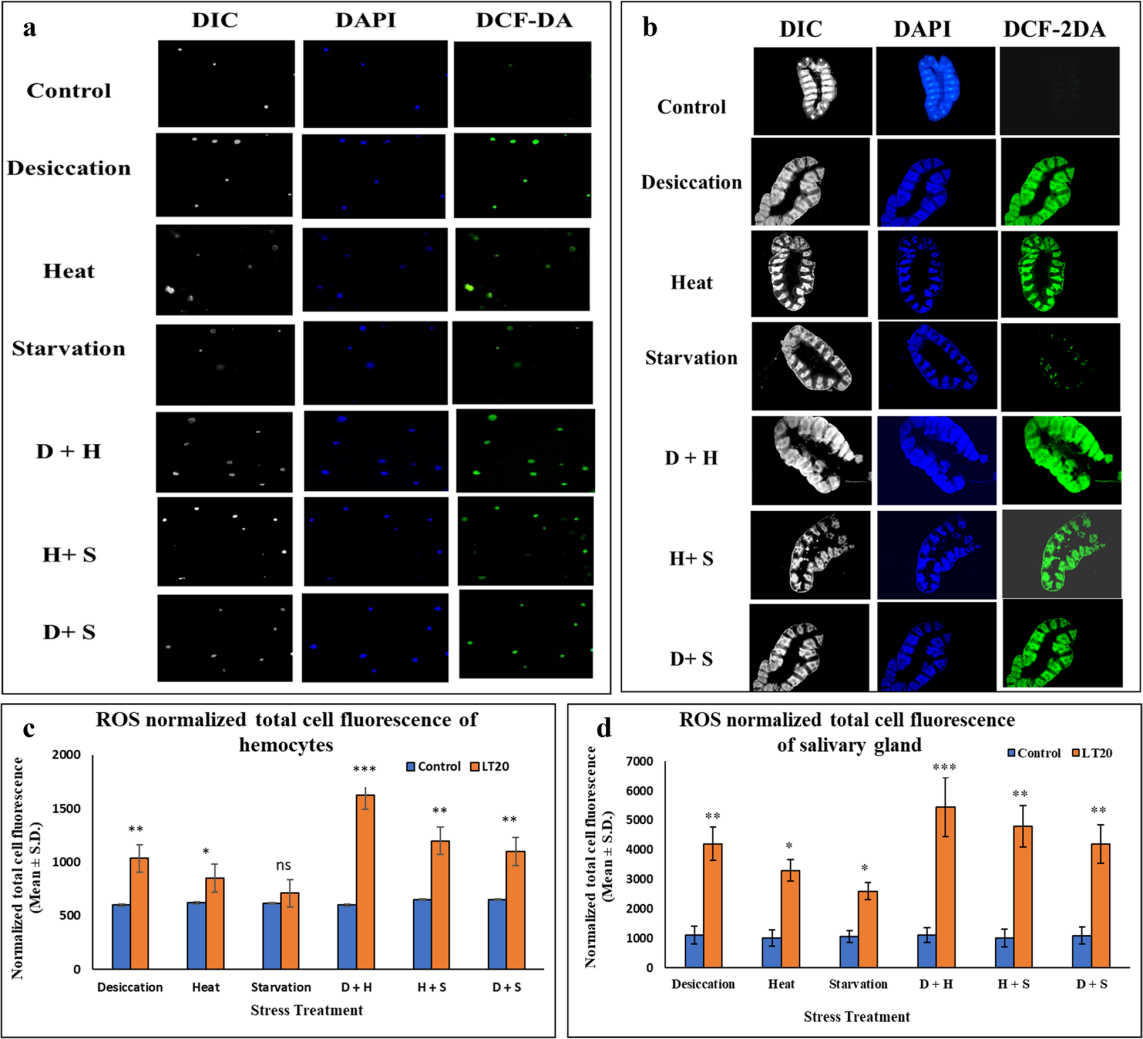
Cellular level visualization of reactive oxygen species (ROS): Fluorescence microscopic images of fluorescent dye 2′,7′-dicholorodihydrofluorescein diacetate (DCF-2DA), 4′,6-diamidino-2-phenylindole (DAPI) and differential interference contrast (DIC) of hemocytes and salivary glands of *C. ramosus* exposed to desiccation, heat, starvation, D + H, H + S, D + S stress treatments compared against control **a** hemocytes (400X) **b** salivary glands (100X) to visualize ROS. Normalized cell fluorescence intensity of **c** hemocytes **d** salivary gland of *C. ramosus* to quantify reactive nitrogen species. ***P < 0.001; **P < 0.01; *P < 0.05: ns- nonsignificant

In fluorescence microscopic analysis, significant changes in ROS generation were noticed in hemocytes and salivary glands of *C. ramosus.* Hemocytes isolated from the treated larvae showed relative increase in green fluorescence intensity compared to the hemocytes isolated from the control larvae of *C. ramosus*. The increase in the intensity of green fluorescence for DCF-DA was significantly higher in the hemocytes isolated from the desiccation stressed larvae than the hemocytes isolated from the starvation and heat stressed larvae. On the other hand, in combinatorial form of stress exposure, hemocytes isolated from the larvae treated with D+H showed significantly higher level of ROS than the other two combinatorial forms. The normalized fluorescence intensity showed expected changes in the treated and control ones. While it was more in desiccation treatment relative to heat and starvation, it was less in H+S and D+S combinatorial treatment compared to D+H treatment. Salivary glands of *C. ramosus* isolated from the treated and the control larvae were also used for florescence microscopic studies. ROS, as quantified by green fluorescence intensity of DCF-DA dye increased in the salivary glands of desiccated larvae as compared to starved and heat stressed larvae. Interestingly, in combinatorial form, salivary glands from D+H exposed samples showed higher degree of fluorescence signal. There was apparent increase in the fluorescence intensity with stress treatment as compared to salivary glands dissected from the control larvae. There was significant increase in normalized cell fluorescence intensity in the salivary glands, sampled from the treated larvae than the control ones (Fig. 3 a-d).

### RNS generation

#### Measurement of nitric oxide radical and nitrite/nitrate concentration

**Fig. 4:**
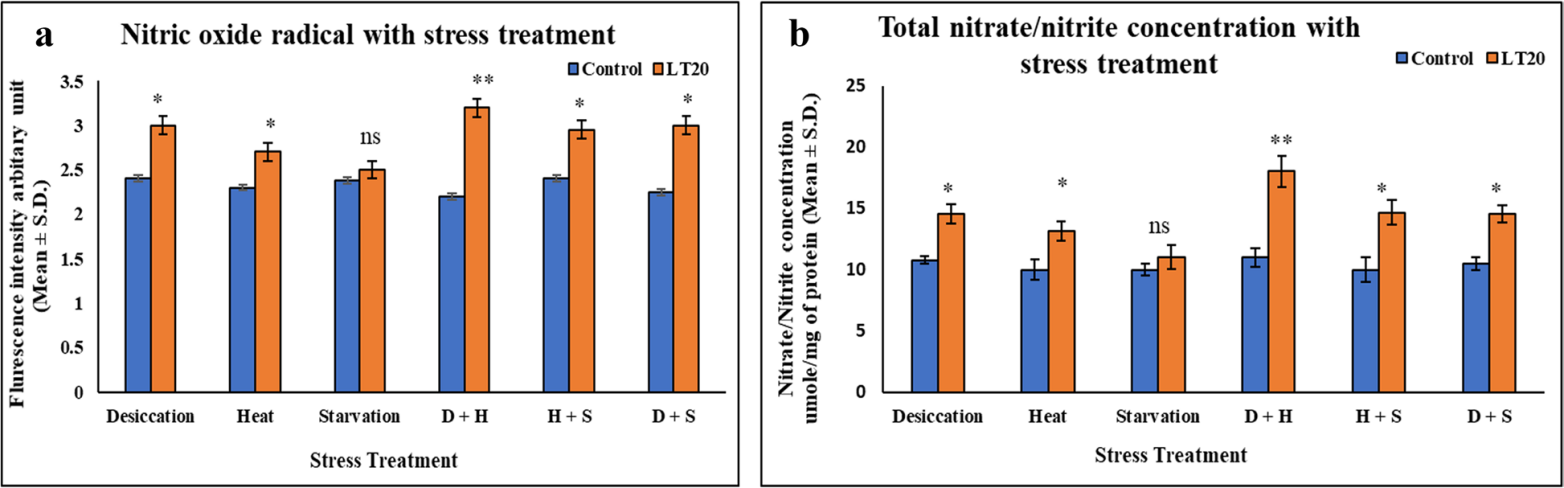
Measurement of reactive nitrogen species (RNS) **a** Determination of Total Nitrate/Nitrite concentration μmole/mg of protein (mean ± SD) in fourth instar larvae of *C. ramosus*. **b** Estimation of level of nitric oxide radical arbitrary unit (mean ± SD) in fourth instar larvae of *C. ramosus*. Values represent mean and the vertical bars represent SD. Data shown are representative of three independent experiments. ***P < 0.001; **P < 0.01; *P < 0.05 indicates significance level

The rate of generation of reactive nitrogen species was higher in the treatment group than that of control. Higher level of reactive nitrogen species, nitric oxide radical and peroxynitrite were found in the treated larvae of *C. ramosus* along with higher ratio of nitrate/nitrite concentration. Singular exposure of DS and HS showed drastic changes as compared to SS treatment. In combinatorial stress response studies, D+H was more vulnerable than H+S and D+S (Fig 4 a-b).

#### RNS Imaging studies

**Fig. 5:**
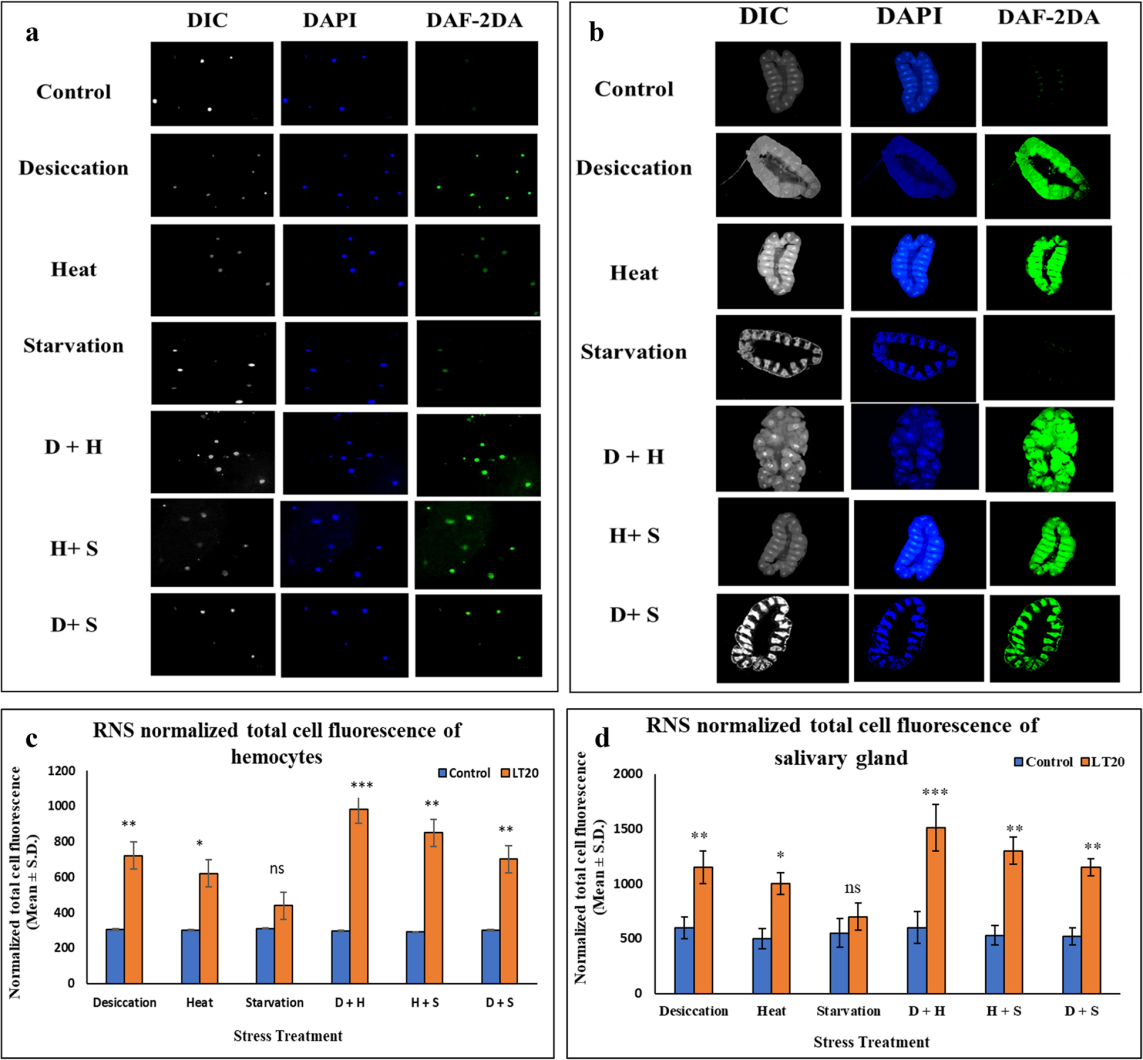
Cellular level visualization of reactive nitrogen species (RNS): Fluorescence microscopic images of fluorescent dye 4,5-diaminofluorescein diacetate (DAF-2DA), 4′,6-diamidino-2-phenylindole (DAPI) and differential interference contrast (DIC) of hemocytes and salivary glands of *C. ramosus* exposed to desiccation, heat, starvation, D + H, H + S, D + S stress treatments compared against control **a** hemocytes magnification (400X) **b** salivary gland (100X) to visualize reactive nitrogen species. Normalized cell fluorescence intensity of **c** hemocytes **d** salivary gland of *C. ramosus* to quantify reactive nitrogen species. ***P < 0.001; **P < 0.01; *P < 0.05 indicates significance level

Hemocytes isolated from the treated larvae of *C. ramosus* showed increase in the intensity of green fluorescence than what was observed in the hemocytes and brain samples isolated from the control larvae. The increase in the intensity of characteristic green fluorescence for DAF-DA in hemocytes and brain samples isolated from the desiccated larvae was more compared to starved and heat stressed larvae. While in combinatorial form, hemocytes isolated from the desiccation coupled with heat stressed larvae showed increase in RNS as compared to other two combinatorial forms of stress exposure. The normalized cell fluorescence intensity showed changes in hemocytes isolated from the treated larvae than that of the control ones. The changes were more conspicuous in DS compared to HS and SS. In combinatorial exposure, it was more significant in case of D+H compared to H+S and D+S. Salivary glands of *C. ramosus* dissected from the desiccated larvae showed more green fluorescence intensity indicative of higher level of RNS than the salivary gland samples taken from the starved and heat stressed larvae. In the case of combinatorial form of stress treatment, salivary glands from the larvae subjected to D+S treatment showed higher fluorescence intensity. There was increase in fluorescence intensity of DAF-DA dye during stress treatment regime as compared to control salivary gland. There was an increase in normalized cell fluorescence intensity in the salivary gland samples obtained from the desiccated as well as desiccation coupled with heat stressed larvae compared to other singular and combinatorial forms of stress exposure (Fig.5 a-d).

### Total RONS concentration and Aconitase enzyme activity

**Fig. 6:**
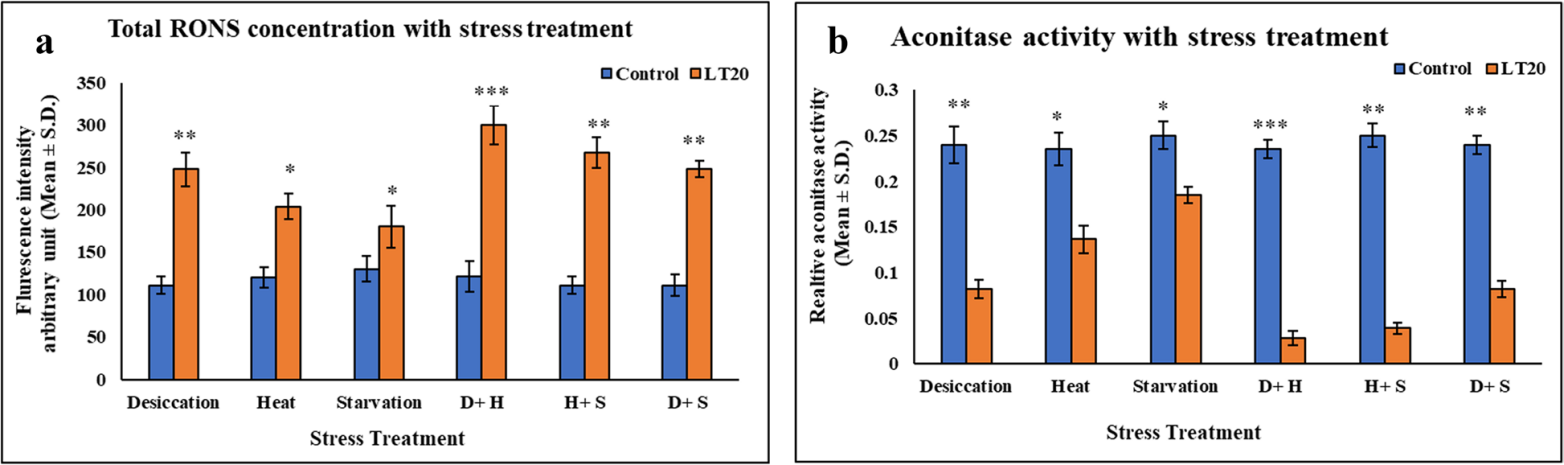
**a** Measurement of total reactive oxygen nitrogen species (RONS) fluorescence intensity arbitrary unit (mean ± SD) **b** Mitochondrial aconitase activity relative to the control with stress treatment (mean ± SD) in fourth instar larvae of *C. ramosus*. Values represent mean and the vertical bars represent SD. Data shown are representative of three independent experiments. ***P < 0.001; **P < 0.01; *P < 0.05 indicates significance level

It was evident from the data that the generation of total RONS was more in DS compared to HS and SS while in combinatorial form, the increase was evident in D+H in contrast to other two combinational form (Fig. 6 a). Stress response studies on aconitase enzyme activity revealed that increase in the level of superoxide radical led to decrease in the catalytic activity of aconitase enzyme. Interestingly, the decrease was more prominent in DS than other individual stressors while in combinatorial treatment, a sharp decline in aconitase activity was noticed in larvae subjected to D+H (Fig. 6 b).

### Antioxidant enzyme activity

**Fig. 7:**
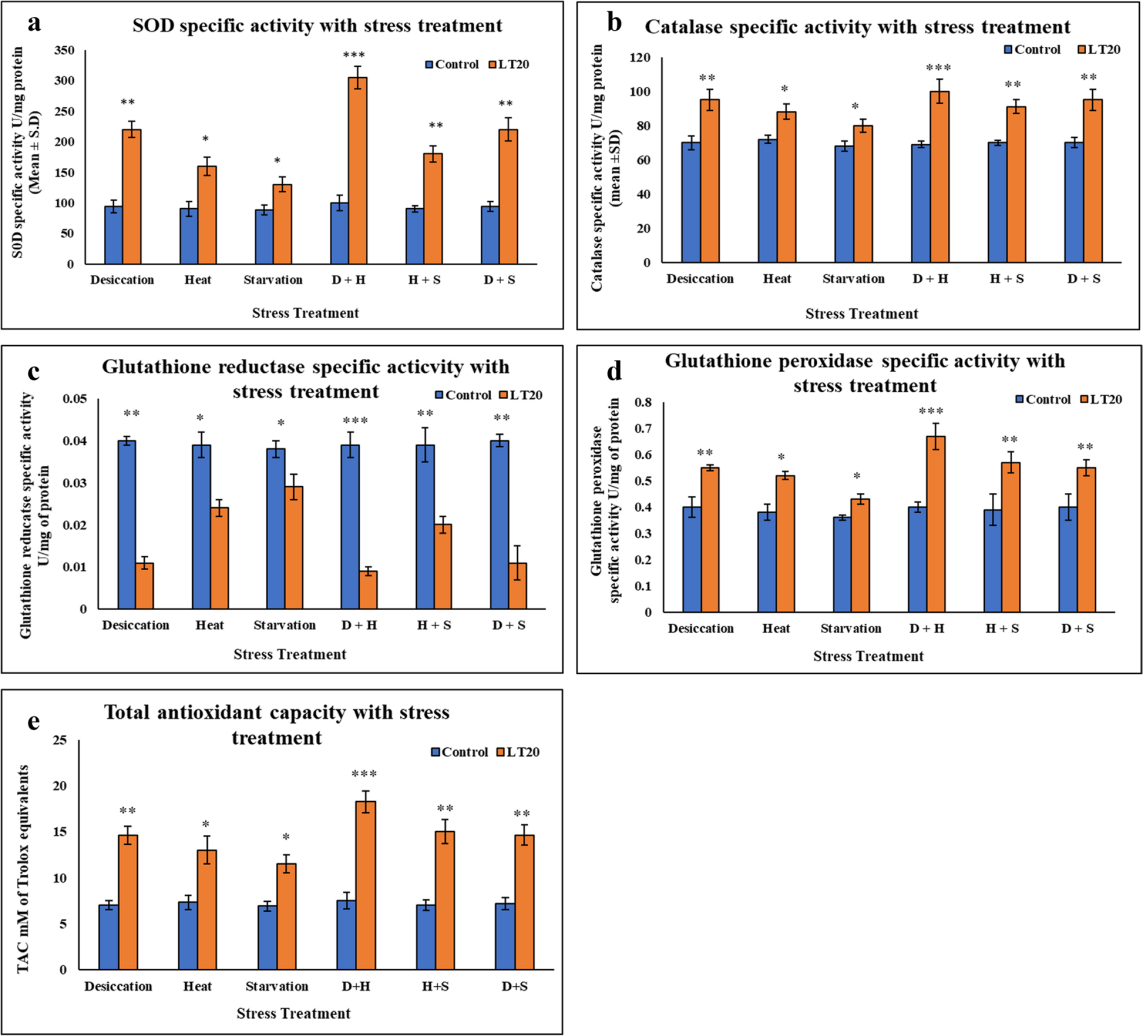
Spectrophotometric measurement of antioxidant enzyme specific activity **a** superoxide dismutase (SOD) **b** catalase activity **c** glutathione reductase **d** glutathione peroxidase of the whole larvae of *C. ramosus* **e** Quantification of Total antioxidant capacity of the whole larvae of *C. ramosus* exposed to desiccation, heat, starvation, D + H, H + S, D + S stress treatments compared with control groups and between treated groups. ***P < 0.001; **P < 0.01; *P < 0.05 indicates significance level

Results of antioxidant enzyme activity revealed that there was an increase in the activity of antioxidant enzymes with all types of stress treatments as compared to control larvae. Levels of SOD activity in larval extract from the treated batch increased significantly compared to that of the control batch. The CAT enzyme activity in whole larval extract showed an increase as compared to basal levels of CAT activity in the respective controls. In the case of GPx, there was an increase in the specific activity after exposure to stressors as compared to control. Unlike SOD and CAT, GR showed decreased specific activity after stress treatment as compared to control. It was evident that the increase in specific activity of SOD and catalase was more in case of DS than that of HS and SS, while in combinatorial form, it was quite evident that the incremental change was prominent in case of D+H compared to other two combinations *viz.* D+S and H+S. The secondary antioxidant enzymes showed reduced activity with respect to stress treatment, apparently because of increase in the catalase activity. Total antioxidant capacity was higher in respective stressor exposed larvae compared to corresponding control larvae (Fig. 7 a-e).

## Discussion

Aquatic fauna are increasingly becoming vulnerable to multiple abiotic stressors, mainly influenced by global warming and climate change. Hence there is an urgent need for robust studies on different eco-indicator species (Wang et al.^2021^). Chironomid midges are well recognized for their role as biomonitoring species of aquatic ecosystem (Nicacio and Juen 2015) and therefore, *C. ramosus* has been chosen for our study due to its remarkable tolerance to abiotic stressors (Bomble and Nath 2019). The present study provides insight to understand the consequences of adverse environmental conditions like desiccation, heat and starvation on *C. ramosus*, when these stressors occur simultaneously as well as in isolation. There were a few seminal findings reporting the generation of oxidative stress due to the effect of different environmental stressors in insects (Thorat et al. 2016, Zhu et al. 2017). Our study has revealed the comparative effect of stressors in singular and combinatorial form in *C. ramosus*. The findings have highlighted the level of RONS generation is stress and combination of stressors specific. We have used only suboptimal stress level which is environmentally relevant (LT_20_) to investigate the synergetic effect of multiple stressors.

The association of heat, desiccation and starvation as stressors has been well studied. Desiccation stress in natural habitat is usually mediated by an increase in ambient temperature and quite often, under these circumstances, organisms undergo nutritional deprivation (Hoffmann and Harshman 1999). In this context, water deficiency, increase in temperature and nutrient deprivation leads to ionic fluctuation which eventually generates reactive species molecules like ROS and RNS. In general, there is a balance between generation of reactive species and scavenging. However, the balance is disturbed under various environmental stress. Poikilotherms are usually exposed to various challenges to survival and reproduction in their environments, and temperature is a critical abiotic factor that causes physiological changes in arthropods. Exposure to temperature stress may lead to generation of excess ROS and lead to oxidative damage in insects (Lalouette et al.^2011^ & Jia et al. 2011, Cui et al. 2011). Deficiency of water leads to ionic imbalance which leads to generation of ROS and ultimately oxidative stress, while transient starvation period leads to generation of oxidative stress in insects (Kumar et al. 2011, Contreras-Porcia et al. 2011 and De Block and Stoks 2008). Most of the previous studies related to organismal response to multiple stress are rather descriptive without providing any inferences towards biochemical, physiological, molecular mechanism for the observed consequences. The present study focused on redox mechanism during the multiple stress regime. This is because ROS generation is one of the most common endpoints irrespective of stress.

The increase in the concentration of oxidative stress markers like MDA, AOPP, carbonyl content indicates oxidative stress (Halliwell and Chirico 1993, Chantal et al. 2004, Locke 1997). Upon stress exposure, it was found that there was significant increase in lipid peroxidation levels as compared to control larvae in *C. ramosus*, as lipids are more susceptible to be attacked by ROS (Halliwell and Chirico 1993). With lipid peroxidation it was also observed that carbonyl content, AOPP levels were also increased in comparison to control larvae. Protein carbonyl content is a marker of protein oxidation, because highly reactive free radical species can oxidize proteins. In this study, after stress treatment, the carbonyl content and AOPP concentration was higher in desiccation than that of individually encountered heat and starvation stressors indicating that in desiccation stress, larvae of *C. ramosus* suffered relatively more oxidative stress. Interestingly in combinatorial form, the level was more in case of desiccation coupled with heat stress than the other two combinatorial form of stress exposures. Increase in MDA concentration in treated larvae as compared to control larvae confirmed the biochemical signature of oxidative stress, although the increase was more evident in heat stress encountered in singular form. In combinatorial form, it was more evident in heat in conjunction with desiccation stress than what could be seen in other combinations.

One of the indicators of oxidative stress generation is the inhibition of enzymatic activity of mitochondrial aconitase enzyme. The rate of aconitase inactivation is directly proportional to increase in the rate of superoxide production in the mitochondrial matrix (Liang et al. 1997; Miwa and Brand 2005). The magnitude of aconitase inactivation under different stress conditions could be correlated with the generation of oxidants, as has been presented in this paper. To best of our knowledge, this is the first report of abiotic stress induced inactivation of aconitase in an aquatic eco-indicator insect like Chironomid midge.

A consequence of adverse environmental stress condition is the generation of RONS leading to oxidative stress hence there are up-regulation of antioxidant enzymes like SOD, Catalase and GPx and down regulation of GR. In the present study, it was evident to observe increased levels of antioxidants and rise in the total antioxidant pool produced by the larvae against abiotic stressors. In arthropods, insects have the capacity to increase antioxidant level to maintain balance in ROS metabolism and to relieve the adverse effect of oxidative stress (Krishnan et al. 2008). Firstly, the activity of three key antioxidant enzymes: SOD, CAT and GPx were assessed, as they form the first line of defense (Ighodaro and Akinloye 2018). SOD converts superoxide anions (O_2_^−^) into less reactive species namely molecular oxygen (O_2_) and hydrogen peroxide whereas CAT and GPx further converts this H_2_O_2_ into H_2_O and O_2_ (Weisiger and Fridovich 1973, Droge 2002). Therefore, as envisaged in our study, substantial increase in the enzymatic activity of SOD, CAT and GPx was found in each stress treatment which confirmed prevailing oxidative stress in theses larvae subsequent to stress exposure. However, we found decline in the GR activity and reduced levels of glutathione in the larvae. Therefore, changes seen in the activity of GPx were very little as compared to control as it required reduced glutathione for its activity. The antioxidant enzyme activity (SOD, CAT, GPx) and total antioxidant enzyme activity (TAC) of *C. ramosus* was found be higher to minimize abiotic stress induced oxidative stress damage. Moreover, the level of antioxidant enzyme activities (SOD, CAT, GR and GPx) and TAC varied significantly after singular and combinatorial stress treatments, indicating increment in the status of oxidative stress when larvae got exposed to different abiotic stressors. In combinatorial form, when larvae were exposed to desiccation with heat stress, the activities of SOD, CAT and GPx were greater than those seen under other combinations. The increase in activity of these three antioxidant enzymes indicated that they may play important role in managing oxidative stress and cellular homeostasis.

In this study, our aim was mainly focused on combinatorial stress responses. Generation of reactive species was more prominent in combinatorial stress treatment as compared to singular stress exposures. Total generation of reactive species was 50% more in case of DS as compared to control, while 30% and 15% more in HS and SS treatments respectively. In combinatorial stress treatment, it was 60% more in D+H, while it was 52% and 50% in H+S & D+S respectively. Therefore, our findings showed that the level of RONS generation was significantly more in case of combinatorial stress treatment depending on the type of combination of stressors. Moreover, the most intriguing finding of the present study was the revelation of changes in RNS level due to nitrosative stress and this is the first report of its kind in *C. ramosus*. When superoxide radical increased it led to the generation of nitric oxide, nitrite and nitrate in a continuum. The biochemical findings were further assessed by fluorescence microscopic analyses, which strongly suggested that the quantum of multiple stress response depended on the type of individual stressors and the kind of combination of stressors.

In summary this study has revealed that RONS generation increases when larvae face combination of multiple stressors, compared to situations when the larvae are exposed to individual stressors. *C. ramosus* larvae showed more tolerance towards starvation and heat stress than to desiccation while in combinatorial form it is more lethal in D+H than other forms of combination i.e., D+S and H+S. RONS generation and survival rate of *C. ramosus* larvae depends upon the type of combination and specificity of stressors. This study provides insight to envisage catastrophic consequences of events of multiple stress exposure in sublethal level. This study will also be useful as an ecosystem indicator in ongoing global concern for climatic changes.

## Materials and Methods

### Rearing and maintainace of model organism

Isofemale line of *C. ramosus* were maintained in the laboratory conditions at 25°C ± 2 exposing them to 14hrs light and 10hrs of dark conditions in the stress biology research laboratory, Department of Zoology, S.P. Pune University as described by Bomble and Nath (2019). Haegele (1975) described a salt medium to simulate pond water for the rearing and for experimentation with *Chironomus* and we used the same for the present study. Photoperiod, maintained in the insectary, mimics the natural condition of Indian fresh water ecosystem where the original stock was seeded. Early fourth instar larvae were used for all the experiments.

### Stress Treatment

Early fourth instar larvae of *C. ramosus* were used for all the experimental work. Larvae were desiccation treated in a desiccator chamber (<5% relative humidity) on a dry tissue paper placed in a glass petri plate. For starvation stress, larvae were kept in petri plates without any nutrients. For heat stress, we kept the larvae in petri plates along with nutrient medium (Bomble and Nath 2019) inside the incubator at 40 °C. For combinatorial stress treatment, larvae were exposed to two different stressors concurrently *viz.* desiccation with heat, heat with starvation, desiccation with starvation. LT_20_ (lethal time required for 20% mortality in the population) values were taken as an end point for each experiment. (Supplementary data, Table: 1-6). In subsequent text, following abbreviations are used *viz.* Desiccation Stress (DS), Heat Stress (HS), Starvation Stress (SS), Desiccation with Heat stress (D+H), Heat with Starvation (H+S), Starvation with-Desiccation (D+S). Since various parameters of stress response manifested starting with LT_20_ doses (for D, H, S, D+S, H+S, D+H, MS), we chose LT_20_ as the base-level parameter for all treatments. LT_20_ values were used for all the experiments carried out to compare between effect of stressors administered either as a singular and or in a combinatorial form. All the experiments were replicated thrice per treatment and were carried out under laboratory conditions.

### Assay of oxidative stress markers

#### Lipid peroxidation (LPO)

The level of lipid peroxidation in the tissue was measured by quantifying the amount of malondialdehyde (MDA) produced as a by-product of lipid peroxidation to check the amount of oxidative stress created in the larvae according to the method given by Bar-Or et al. (2001). A standard graph was plotted using 2 mM MDA standard and appropriate dilutions for calculating unknown concentrations of MDA from samples. 100 μL of 20% trichloroacetic acid and 50 μL of the sample were mixed and centrifuged at 15,000 g for 10 min at 4 ˚C. Then, the supernatant was mixed with 100 μL of 0.8% TBA reagent and re-incubated at 100 ˚C for 60 min before reading absorbance at 535 nm. The concentration of MDA in the sample test was calculated in terms of umol/mg.

#### Protein carbonyl content

Protein carbonyl content was measured for its ability to react with DNPH and the resultant protein-hydrozone was quantified spectrophotometrically at 360nm following the manufacturer protocol of protein carbonyl colorimetric assay kit (Cayman chemical, Item No. 10005020). Larvae were homogenised in ice-cold buffer (50 mM phosphate, 1 mM EDTA, pH 6.7), centrifuged at 10000 g for 15 min at 4 °C retaining supernatant for further analysis and treated with 10% streptomycin sulphate to remove nucleic acids. Assay mixture consisting of sample, DNPH, 2.5 M HCL, 20% TCA were centrifuged 10000 g for 10 min at 4 °C. Pellet resuspended in 1 ml of 1:1 ethanol/ethyl acetate mixture was centrifuged at 10000 g for 10 min at 4 °C, repeated twice. Pellet resuspended in 500ul of guanidine hydrochloride was centrifuged at 10000g for 10 min at 4 °C. Absorbance of supernatant was measured at 360 nm by UV spectrophotometer.

#### Advanced oxidative protein products

Advanced oxidative protein products were measured as described by Witko-Sarsat et al. (1996). Control and treatment samples were incubated with potassium iodide for 30 min on rocking shaker at 23±1 °C (RT). Absorbance was spectrophotometrically measured at 340 nm after addition of ethyl acetate, were readings normalized with Chloramine T standard plot.

### Assay for ROS generation

#### Measurement of superoxide radicals

After stress treatment, the larvae were homogenized in 1X phosphate-buffered saline (PBS) and centrifuged at 5000rpm at 4 °C for 10 min. The supernatants obtained were incubated in the dark in 2′,7′-dicholorodihydrofluorescein diacetate (DCF-DA; Invitrogen, D399) solution for 20 min for the detection of O_2_^·−^. Post incubation, the supernatants obtained were measured by fluorimetry at dye-specific excitation/emission wavelengths. DCF-DA dye solution was run as standard and data was represented as arbitrary fluorescence units.

#### Measurement of Hydrogen peroxide

The amplex red hydrogen peroxide assay kit (Invitrogen-A22188) was used to determine the concentration of hydrogen peroxide in control and treatment samples as per manufacture’s protocol. Treated and control larvae at respective time points were homogenized in assay buffer solution, centrifuged at 10000 g for 15 min at 4 °C. Supernatant was used in reaction mixture containing dye with HRP. The absorbance was measured at 560 nm using microplate reader and readings, were normalized with standard plot of H_2_O_2_.

### Assay for RNS generation

#### Measurement of total nitrate/nitrite concentration

Total nitrate/nitrite concentration was measured by using total nitrate/nitrite colorimetric assay kit (Cayman chemical, 780001) by following manufacture’s protocol. Control and treated larvae were homogenized in buffer, centrifuged at 10000 g for 20 min at 4 °C. Supernatant was used for further procedure. The nitrate/nitrite concentration was determined by using standard plot of nitrate and nitrite concentration.

#### Measurement of Nitric oxide radical

The concentration of nitric oxide radical was determined by using fluorescent dye 4,5-Diaminofluorescein diacetate (DAF-2DA, Abcam, ab145283) specific for reactive nitrogen species. After stress treatment, the larvae were homogenized in 1X phosphate-buffered saline (PBS) and centrifuged at 5000 rpm at 4 °C for 10 min. The supernatant was incubated in the dark in DAF-DA dye solution for 20 min. Post incubation, the supernatants were measured by fluorimetry at dye-specific wavelengths (Ex/Em= 491/513). DAF-DA dye solution was run as standard and data was represented as arbitrary fluorescence units.

### Estimation of total RONS concentration

Total RONS generation was quantified by using total RONS detection kit (Enzo chemical, ENZ-51011) in salivary gland cells of *C. ramosus* larvae of control and treatment group by following manufacture’s protocol. Salivary glands were dissected in Schneider’s insect medium and single cell suspension was prepared, centrifuged at 3000 rpm for 5 min at 4 °C. Supernatant was discarded and cells were resuspended in assay buffer solution for quantification of RONS level in control and treated larvae. Absorbance was taken on microplate reader (FLUOstar Optima, BMG Labtech, Germany) and data were represented as arbitrary fluorescence units.

### Cellular level study to investigate status of reactive species

Haemocytes from larvae of *C. ramosus* were isolated from the control and treatment group by capillary method (Maier W. 1969). Hemolymph was collected in centrifuge tubes and was centrifuged at 3000 rpm for 5 min at 4 °C. Cells (1× 10^4^ cells per 100 larvae) were resuspended in 1X PBS. Whole salivary glands from treated and control larvae were dissected. Isolated hemocytes and salivary glands were used for microscopic visualization of RONS.

#### Assay for visualization of ROS generation

Intracellular ROS was analysed by florescence microscopy using 2′,7′-Dichlorodihydrofluorescein diacetate (DCF-DA; Cayman). The hemocytes and dissected salivary gland from control and treated group were incubated with DCF-DA dye for 20 min in the dark, hemocytes were centrifuged at 3000 rpm for 5 min at 4 °C, followed by counter staining of DAPI for 5 min in the dark. The samples were observed under Fluorescence microscope (Carl Zeiss, Germany), using appropriate filters for DCF-DA (Ex/Em= 492–495/517–527) and DAPI (Ex/Em = 358/461). We have quantified the normalized values of fluorescence intensity using software ‘Image J’. Differential Interference Contrast (DIC) images were also provided for better visualization of cell and tissue samples.

#### Assay for visualization of RNS generation

The isolated haemocytes and dissected salivary gland from control and treated group were incubated with 4,5-Diaminofluorescein diacetate (DAF-2DA; Abcam) binds to nitric oxide radical for 15 min in the dark, hemocytes were centrifuged at 3000 rpm for 5 min at 4 °C, followed by counter staining of DAPI for 5 min in the dark and observed under fluorescence microscope (Carl Zeiss, Germany), using appropriate filters for DCF-DA (Ex/Em = 491/513) and DAPI (Ex/Em = 358/461). For determining the normalized cell florescence intensity, three different fields were used to quantify florescence intensity of individual images of control and treatment group of hemocytes as well as salivary glands. All the experiments were repeated independently.

### Estimation of aconitase enzyme activity

Mitochondria were isolated from early fourth instar larvae of *C. ramosus* from control and treated group modifying procedure described by Wen et al. (2016). Aconitase activity was measured spectrophotometrically (Thermo scientific instruments) while nicotinamide adenine dinucleotide phosphate (NADPH) formation was monitored at 340 nm following coupled assay described by Gardner (2002), serving as sample to quantify aconitase enzyme activity. The sample was added to assay buffer (50 mM Tris–HCl pH 7.4, 0.6 mM MnCl_2_, 5 mM sodium citrate, 0.2 mM NADP^+^, 0.1% v/v TritonX-100 and 0.4 units/ml isocitrate dehydrogenase {Sigma} pre-equilibrated to 30 °C). Each sample was assayed in quadruplicate, readings were taken at 15-s intervals over 7 min, and the resulting linear slopes were averaged to give a measurement of aconitase activity for that sample.

### Measurement of anti-oxidant enzymes

#### Preparation of homogenate for antioxidant assay

Larvae from control and treated group were homogenized in chilled protein extraction buffer.-consisting of 1 mM phenyl methyl sulfonyl fluoride (PMSF), 1 mM ethylenediaminetetraacetic acid (EDTA), 50 mM phosphate buffer (pH 7.2), 0.1% TritonX-100. Larvae were homogenized and centrifuged at 14000 rpm for 30 min at 4 °C and the enzyme activity was determined from this homogenate followed by quantification of protein by Bradford method, using bovine serum albumin as standard.

#### Superoxide dismutase (SOD) assay

The specific activity of super oxide dismutase (EC 1.15.1.1) was determined by measuring its ability to inhibit the photochemical reduction of nitro blue tetrazolium chloride (NBT), described by Beauchamp and Fridovich (1971). The reaction mixture consisted of 100 mM KPO_4_ buffer pH7.8, 0.01 μM EDTA, 65 mM L-methionine, 750 μM NBT, 2 mM riboflavin and 50 μL of enzyme extract in a total volume of 3 mL. Riboflavin was added at the end and the tubes were mixed by shaking. Two sets of the above reaction mixture were made; one kept in light (20 W) while other in dark for 30 min. Mixtures without enzyme extract were similarly kept under light and dark and used as controls. Absorbance was measured at 560 nm. One unit of SOD activity (U) was defined as the amount of enzyme required to cause 50% inhibition of photo reduction rate of NBT. Results were expressed as unit activity (U)/mg of protein

#### Catalase (CAT) assay

The activity of catalase (EC 1.11.1.6) enzyme was measured as described by Aebi (1983). Reaction mixture consisted of 100 mM phosphate buffer pH 7.0, 20 mM H_2_O_2_, 50 μL enzyme extract in a total volume of 1mL in quartz cuvettes. The decrease in the amount of H_2_O_2_ was monitored by taking absorbance at 240 nm at the interval of 30-s for 3 min on spectrophotometer. One unit of enzyme was defined as the amount of enzyme required to convert 1mol of H_2_O_2_ to product in 1-s. The results were expressed in unit activity (U)/ mg of protein.

#### Glutathione reductase (GR) assay

The activity of glutathione reductase (EC 1.8.1.7) was determined as described by Goldberg and Spooner (1983). Assay mixture consisted of 100 mM phosphate buffer pH 7.2, 0.17 mM NADPH, 0.5 mM EDTA, 2.2 mM oxidized glutathione and 100 μL of enzyme extract in a total volume of 1 mL. All components were mixed properly and rate of oxidation of NADPH was monitored up to 5 min at intervals of 30-s by measuring absorbance at 340 nm. The enzyme activity was calculated in terms of U/mg of protein.

#### Glutathione peroxidase (GPx) assay

Activity of Glutathione peroxidase (EC 1.11.1.9) was determined according to the method of Lawrence and Burk (1976). For this assay, the reaction mixture consisted of 100 mM KPO4 buffer pH-7.0,1 mM NaN_3_, 0.2 mM NADPH, 1U GR, 1 mM GSH, 0.25 mM H_2_O_2_, 1 mM EDTA and 100 μL of enzyme extract in a total volume of 1mL. Absorbance was measured at 340 nm for 5 min at an interval of 30-s. Enzyme activity was measured in terms of U/mg of protein.

### Total Antioxidant Capacity

Total antioxidant capacity of samples was determined on the basis of its ability to inhibit oxidation of ABTS (2,2'-azino-di-3-ethylbenzthiazoline sulphonate) by metmyoglobin and the absorbance was measured at 405 nm using manufacturer’s protocol (Cayman, 709001). The capacity of the antioxidants in the sample to prevent ABTS oxidation is compared with that of Trolox, a water-soluble tocopherol analogue, and is quantified as molar Trolox equivalents. Control and experimental larvae of *C. ramosus* were homogenized in 100 μL of extraction buffer (provided with kit), and then centrifuged at 12000 rpm for 15 min at 4 °C while supernatants were used for further experiments. Standard curve was prepared using different concentrations of Trolox for calculating total antioxidant capacity of samples.

### Statistics

To determine LT_20_ values, log probit analysis was carried out for each experiment. Analysis of variance was carried out to find out the significant difference between each control and treatment group and also within a treatment group. All the experimental data were represented in the form of Mean ± SD, values obtained were subjected to one-way ANOVA using SPSS, version 22.0. The post hoc test was used for the statistical comparison between the groups and Tukey's multiple comparisons test was carried out to determine statistical significance within the groups. The level of significance was indicated as; ***P<0.001; **P<0.01; *P<0.05 and ns: not significant.

## Data Availability

The data and materials that support the findings of this study are available from the corresponding author upon request.

## Abbreviations

LT_20_: Lethal time required to kill 20% larvae
DS: Desiccation stress
HS: Heat stress
SS: Starvation stress
D + H: Desiccation with heat stress combined
H + S: Heat with starvation stress combined
D + S: Desiccation with starvation stress combined
MS: Multiple stress
s: Second
min: Minutes
ROS: Reactive oxygen species
RNS: Reactive nitrogen species
RONS: Reactive oxygen nitrogen species
SOD: Superoxide dismutase
CAT: Catalase
GR: Glutathione reductase
GPx: Glutathione peroxidase
O_2_^−^: Superoxide anions
O_2_: Molecular oxygen
H_2_O_2_: Hydrogen peroxide
GSH: Reduced glutathione
H_2_O: Water
TAC: Total antioxidant capacity
MDA: Malionaldehyde
AOPP: Advanced oxidative protein products
LPO: Lipid peroxidation
TBA: Thiobarbituric Acid
TCA: Trichloroacetic acid
EDTA: Ethylenediaminetetraacetic acid
DNPH: 2,4-Dinitrophenylhydrazine
HCL: Hydrochloric acid
PBS: Phosphate-buffered saline
DCF-DA: 2′,7′Dicholorodihydrofluorescein diacetate
HRP: Horseradish per- oxidase
DAF-2DA: 4,5-Diaminofluorescein diacetate
DCF-DA: 2′, 7′-Dichlorodihydrofluorescein diacetate
DAPI: 4′,6-Diamidino-2-phenylindole
NADPH: Nicotinamide adenine dinucleotide phosphate
MnCl_2_: Manganese(II) chloride
PMSF: Phenyl methyl sulfonyl fluoride
rpm: Rotation per minute
ANOVA: Analysis of Variance
NBT: Nitro blue tetrazolium chloride
KPO_4_: Potassium phosphate
NaN_3_: Sodium azide
ABTS: 2,2'-Azino-di-3-ethylbenzthiazoline sulphonate
